# Transmissible Gastroenteritis Virus (TGEV) and Porcine Respiratory Coronavirus (PRCV): Epidemiology and Molecular Characteristics—An Updated Overview

**DOI:** 10.3390/v17040493

**Published:** 2025-03-28

**Authors:** Monika Olech, Marta Antas

**Affiliations:** 1 Department of Research Support, National Veterinary Research Institute, Al. Partyzantów 57, 24-100 Pulawy, Poland; 2 National Veterinary Research Institute, Al. Partyzantów 57, 24-100 Pulawy, Poland; antas.marta@gmail.com

**Keywords:** TGEV, PRCV, coronavirus, pig, epidemiology, mutation, virulence

## Abstract

Transmissible gastroenteritis virus (TGEV) and porcine respiratory coronavirus (PRCV) are enveloped, single-stranded RNA viruses belonging to the genus *Alphacoronavirus* in the family *Coronaviridae*. PRCV, a TGEV mutant with a spike(S) gene deletion, exhibits altered tissue tropism. TGEV replicates mainly in the intestines and causes severe diarrhea and high mortality in piglets, whereas PRCV replicates mainly in the respiratory tract. PRCV causes mild or subclinical respiratory infections but may contribute to respiratory disease syndrome in pigs infected with other respiratory pathogens. As PRCV and TGEV continuously evolve, monitoring these viruses is important for disease prevention and control. In this review, we provide updated information on the prevalence and genetic characteristics of TGEV/PRCV and their phylogenetic relationships. We also discuss the impact of mutations, deletions and recombination on the virulence and tissue tropism of TGEV/PRCV and highlight the possible zoonotic potential of these viruses.

## 1. Introduction

Transmissible gastroenteritis virus (TGEV) and porcine respiratory coronavirus (PRCV) are enveloped, single-stranded positive-sense RNA viruses belonging to the genus *Alphacoronavirus* in the family *Coronaviridae*. TGEV was first identified in 1946 by Doyle and Hutchings in the U.S. and was a major disease of pigs worldwide until the late 1980s, when a mutant variant of TGEV called porcine respiratory coronavirus emerged and spread widely worldwide. Infection with TGEV or PRCV induces antibodies that neutralize both viruses, so PRCV infection causes immune cross-protection against TGEV, and this phenomenon has changed the global epidemiology of TGEV. In addition, PRCV-infected sows and gilts can induce milk-derived immunity to TGEV, so maternal antibodies can provide piglets some level of protection against TGEV in the first weeks of life [1]. PRCV is a natural mutant of TGEV, first isolated in Belgium in 1984 [2], which has a characteristic deletion at the 5′ end of the S (spike) gene. TGEV replicates in both the intestines and respiratory tract, whereas PRCV replicates mainly in the upper respiratory tract and lungs, with limited replication in intestinal epithelial cells. Therefore, for TGEV detection, mainly feces, oral fluids and intestinal contents are used, whereas nasal or lung swabs are used for PRCV detection.

PRCV usually causes subclinical respiratory infections, but it may contribute to respiratory disease syndrome in pigs infected with other respiratory pathogens [3,4]. Previous studies have shown that co-infection of PRCV and pathogens such as porcine reproductive and respiratory syndrome virus (PRRSV), swine influenza virus and *Bordetella bronchiseptica* can cause more severe pneumonia than single infections can cause. PRCV infection increases PRRSV replication [5]. PRCV spreads rapidly because of the respiratory nature of the infection, whereas TGEV is transmitted mainly via the fecal-oral route. TGEV causes acute diarrhea with significant morbidity in pigs of all ages and high mortality in pigs less than two weeks old. Therefore, disease caused by TGEV is notifiable by the World Organization for Animal Health (WOAH) [6]. However, animals in herds simultaneously infected with PRCV and TGEV show less severe clinical signs [7].

Four major structural proteins have been identified in PRCV and TGEV: glycoprotein S (spike), envelope (E), nucleocapsid (N) and membrane protein (M). The M, N and E proteins are identical in PRCV and TGEV. The differences between TGEV and PRCV are found mainly in protein S. Only protein S contains epitopes that induce virus-neutralizing antibodies. The S glycoprotein contains four major antigenic sites, A, B, C and D, located at the N-terminus of the mature S protein. PRCV lacks one or two antigenic epitopes (C and D), which allows differentiation between TGEV and PRCV when monoclonal antibodies to these epitopes are used [8,9,10]. Conventional serological tests such as virus neutralization, indirect fluorescent antibodies, agar gel precipitation, radioimmunoprecipitation, indirect immunoperoxidase and ELISA tests based on polyclonal TGEV antibodies cannot distinguish between TGEV and PRCV because virus-neutralizing antibodies mainly target the A antigenic site, which is conserved in both TGEV and PRCV [11]. Therefore, PRCV was initially confused with TGEV because PRCV-infected pigs were positive in diagnostic tests for antibodies to TGEV. Originally, the diagnosis of PRCV was based on the diagnosis of respiratory disease in pigs with high titers of antibodies to TGEV in which no clinical intestinal lesions were observed. The virus infections can be distinguished serologically by a competitive ELISA using two monoclonal antibodies. The first antibody is directed against the D antigenic site in the S protein, which is deleted in PRCV but present in TGEV. The second monoclonal antibody binds to the A antigenic site of both viruses [10,12,13]. However, it should be noted that serological cross-reactivity between TGEV and PRCV can be significant, even when blocking ELISA tests are used. The occurrence of false positives or inconclusive reactions that are difficult to interpret makes the diagnosis of PRCV and TGEV problematic [10,11,14]. For this reason, it would be best to use such ELISA tests to identify TGEV-infected herds rather than individual TGEV-infected individuals [11]. RT-PCR, real-time RT-PCR, in situ hybridization and multiplex microarray hybridization using primers targeting the S’ region of the S gene covering the deletion region in PRCV are also used to differentiate between PRCV and TGEV [15,16,17].

Since TGEV and PRCV are not considered significant swine pathogens, routine screening for these viruses does not take place. However, these viruses exhibit a high degree of mutation and recombination, which can promote the emergence of new strains with altered biological characteristics, including altered tropism, virulence and host specificity. Therefore, the authors believe that these viruses should be given more attention. Systematic monitoring and surveillance of these viruses are important for disease prevention and control. In this review, we provide updated information on the prevalence and genetic features of TGEV/PRCV and their phylogenetic relationships. We also discuss the impact of mutations, deletions and recombination on the virulence and evolution of TGEV/PRCV. This information improves the understanding of the current status of TGEV and the PRCV.

## 2. Epidemiology of TGEV and PRCV

### 2.1. TGEV in Europe

Outbreaks of TGEV in Europe are rare. Therefore, TGE gastroenteritis is not as prevalent in Europe as it was in the past. In 1984, an increased number of TGEV seropositive animals were reported in Belgium. TGEV-specific antibodies were detected in 7.5% (12/160) of Belgian pigs from 14.8% (12/81) of farms [18], but these data are from the 1990s, and current data are lacking. Outbreaks of TGEV were observed on Hungarian farms, where diarrhea, vomiting and emaciation in infected animals were observed. Serological and RT-PCR results from 2014 revealed that 5/14 herds were infected with TGEV [19]. However, a nationwide serological survey conducted more recently in Hungary using samples collected between 2015 and 2016 revealed that only 1 of 908 (0.1%) serum samples were serologically positive for TGEV [20]. A recent study in southern Italy reported that the seroprevalence of TGEV in the swine population was 5.5% [21], whereas a recent study in Poland reported that 2.2% (18/828) of the pig sera tested were serologically positive for TGEV, and TGEV-positive samples were detected in only three of the 13 Polish regions tested [14]. The percentage of samples positive for TGEV was very low in Spain when samples collected from 2017 to 2018 were examined. TGEV RNA was detected in only 6 of 215 (2.8%) samples with diarrhea [22]. However, TGEV RNA was not detected in any of the fecal samples collected between 2017 and 2019 in Spain [23,24]. TGEV RNA was also not detected in any of the rectal swabs taken from 160 pigs with diarrhea from Slovakia [25] or in any of the 221 fecal samples collected between 2021 and 2024 in Poland [14]. TGEV has never been detected in Denmark [26].

Antibodies against TGEV were found in one of 134 wild boars tested in the Czech Republic, and antibodies were detected via the indirect fluorescence antibody test (IFAT) [27]. In Germany, 1.59% of samples from wild boars had antibodies against TGEV [28], whereas in Italy, the TGEV seroprevalence for samples collected from wild boars between 2016 and 2027 was 0.67% [29]. In Croatia, 0.4% of serum samples collected from wild boars between 2005 and 2010 had antibodies to TGEV [30]. In contrast, in Finland, all 303 tested samples taken from wild boars were serologically negative for TGEV [31]. Antibodies against TGEV were also not present in 178 serum samples taken from wild boars in Slovenia [32].

### 2.2. TGEV in America and Asia

A total of 2263 serum samples originating from five- to six-month-old pigs from Quebec were tested for TGEV, and 7.2% of the serum samples were seropositive [33]. Between 2008 and 2016, 29,397 clinical samples from the U.S. were tested by real-time RT-PCR, and 2.3% of them tested positive for TGEV. In 2008, the percentage of positive samples was 4%. This value increased to 6.8% in 2010 and then decreased to less than 0.1% in 2014. Since 2006, only the TGEV genotype variant has been detected in the US [34]. Recently, a US study revealed that TGEV RNA was not detected in any of the 1245 lung samples tested [35]. The authors speculated that the decline in TGEV incidence was related to the introduction of PEDV into the U.S. and increased biosecurity.

In 2014, TGEV infection was reported in an Argentine pig from a small herd, where 2.3% morbidity was observed. Three clinical cases of TGEV were reported in Argentina between 2010 and 2015. The TGEV antigen was detected in all of the the animals via immunohistochemistry [36]. Antibodies against TGEV were also recently detected in 3.4% (3/87) of collared peccaries in this country, but antibodies against TGEV were not detected in 160 samples taken from the wild boars tested [37]. Antibodies against TGEV/PRCV were not detected in 22 wild boar samples originating from the Saskatchewan region of Canada [38].

Since 2003, TGEoutbreaks have been reported in Cuba [39]. In 2008, TGEV was found in Cuba in 10% of suckling piglets suffering from diarrhea [40].

In Japan, the incidence of TGEV has declined, and only eight outbreaks were reported between 2001 and 2007 [41]. In 2010, an extensive study performed by Miyazaki et al. revealed that antibodies against TGEV were detected in 14.4% of 2703 tested serum samples. Infections were detected in pigs from 29.6% of the farms and three of the six Japanese regions studied [41]. No antibodies against TGEV were detected in 2008, when 112 Japanese serum samples were tested. Moreover, TGEV genetic material was not detected in any of the tested nasal swabs, feces or organ samples [3]. Antibodies against TGEV were also not present in any samples tested from Turkey [42] or the West Indies, where 309 pig samples were tested [43].

A recent study performed by Kim et al. [44] in South Korea revealed that the seroprevalence of TGEV was 4.3% (15/350). However, TGEV RNA was not detected in any clinical samples when RT-qPCR was used. A previous study performed in South Korea by Oh et al. revealed that out of 1295 finisher sera, 64 (4.9%) were positive for TGEV. Among the 69 samples collected from wild boars, not one was TGEV positive [8]. No TGEV outbreaks have been reported in Korea since 2018 [44].

The prevalence of TGEV in pigs in China has been low in recent years. However, some TGEV strains are still prevalent in southern and northern China [45,46,47]. In China, TGEV has been reported since 1958 and has been present in all provinces. The number of TGEV infections exceeded 100,000 between 2008 and 2010, with 366,000 and 159,000 infections reported in 2011 and 2012, respectively. TGEV infection is present in almost all of the Chinese provinces, with prevalence rates ranging from 1% to as high as 95% [48]. The rate of TGEV infection in 2013 and later to 2013 was 12%. The overall detection rate of TGEV RNA in clinical samples collected in 2015 from pigs with diarrhea was 18.2% (8/44) [49]. In contrast, a study of 2987 clinical samples collected between 2012 and 2018 revealed a low frequency (0.7%) of TGEV RNA detection in China [50]. A recent study involving 1791 clinical samples from China (2021–2023) confirmed that the detection rate of TGEV RNA is very low (<1.0%) [51]. In China, the incidence of virulent strains of TGEV has been reported. In 2020, the JS2012 strain was isolated [45]. An analysis of recombination revealed that the TGEV JS2012 strain is a natural recombinant strain. Experimental infection with this strain caused 100% mortality in a group of newborn piglets (n = 5) compared to the control group (n = 5), highlighting the strong pathogenicity of this virus. In 2016, the Chinese strain HQ2016 was isolated, and typical clinical signs and pathological and histological alterations associated with TGEV were observed in piglets inoculated with this strain [52]. In 2021, a new highly pathogenic TGEV strain named SC2021 was isolated in China. This strain had an 81 nt deletion in the ORF3a gene, including the start of ORF3a, which resulted in the loss of nonstructural protein 3a. Compared with TGEV SC-Y-infected piglets, newborn piglets orally infected with TGEV SC2021 presented more severe watery diarrhea and faster mortality. In addition, macroscopic and microscopic changes were observed in the lungs of TGEV SC2021-infected piglets, suggesting that the absence of the ORF3a protein may be the cause of lung injury [53].

Main gaps and challenges:

1. Lack of routine testing: While TGEV is considered rare in most regions, its true prevalence remains uncertain due to insufficient surveillance.
2. Misdiagnosis: Serological tests do not always distinguish PRCV from TGEV, leading to potential misinterpretation of results. TGEV shares clinical signs with other enteric diseases, such as porcine epidemic diarrhea (PED) and rotavirus infections, making them clinically indistinguishable.
3. Impact of PRCV on TGEV prevalence: The emergence of PRCV has been linked to a decrease in the number of animals infected with TGEV, but the exact mechanisms behind this decline require further study.
4. Environmental stability: More research is needed on how long TGEV survives under different environmental conditions. TGEV is often associated with winter outbreaks, but the exact environmental or management factors affecting seasonality require further research.
5. Wildlife reservoirs: The role of wild boars in the spread of TGEV is not well examined.
6. There is a lack of epidemiological models for TGEV spread that integrate key factors (e.g., environmental conditions, wildlife interactions, virus evolution, biosecurity).

### 2.3. PRCV in Europe

On the basis of samples collected from 1985 to 1986, 58.8% of Danish pigs were found to have antibodies to PRCV [54]. A total of 90.6% (145/160) of Belgian pig sera collected in 1990 were positive for PRCV [18]. A seroprevalence rate of 75–80% in pigs in Denmark was reported by Have in 1990 (1990) [26]. A recent study using samples collected between 2021 and 2023 also revealed a very high seroprevalence (74.9%) (191/255 samples) of PRCV in this country [4]. A high PRCV seroprevalence of 65% was also recently reported in pigs from Slovenia [55]. PRCV RNA was detected in 48% of nasal swab samples from pigs with respiratory disease collected between 2017 and 2019 in Spain [56]. A lower seroprevalence of 26% was reported in Norway [57], whereas in Hungary, 15.42% (139/908) of tested samples were PRCV seropositive when 908 sera collected from 2015 to 2016 were tested via an immunofluorescence test [20]. A survey in Poland revealed that 12.2% (101/828) of the tested sera were PRCV-antibody positive. Serologically positive samples were found in all the voivodships studied, indicating that PRCV is widely present throughout Poland. However, PRCV genetic material was less frequently detected in this country. PRCV RNA was detected in 7.6% (21/277) and 4.5% (10/221) of the 277 nasal swab samples and 221 fecal samples, respectively [14]. The detection of PRCV in feces was surprising, as negligible amounts of this virus are observed in feces. This may be due to the presence of specific deletions in the S gene in Polish PRCV strains, which is unusual for other PRCV strains found worldwide [14]. As noted previously, some PRCV-infected pigs can excrete the virus in their feces [15]. PRCV can be detected in the small intestine, but the virus does not replicate in villi epithelial cells [58]. A PRCV strain AR310 from the U.S. was the first strain isolated from the small intestine [59].

A recent study in southern Italy revealed that the PRCV seroprevalence in the pig population was very low (0.9%) [21]. Low PRCV seroprevalence was also detected in wild boars. In Slovenia, the PCRV seroprevalence in these animals was estimated at 3% (5/178) when samples from the 2003/2004 hunting season were examined [32]. A higher seroprevalence, estimated at 7.87%, was observed in Germany, where 1221 samples from wild boars were examined [28]. Low seroprevalence rates, 0.9% and 0.7%, were obtained in samples collected in 2016–2017 from wild boars in Italy [21] and in samples collected in 2005–2010 from wild boars in Croatia [30]. In Finland, none of the 303 samples collected from wild boars were serologically positive for PRCV [31].

### 2.4. PRCV in Asia and America

PRCV was first described in Japan in 1996 [60]. The high seroprevalence of PRCV (90.18%) in Japan was previously reported by Usami et al. [3] when 112 serum samples were tested. In 2010, Miyazaki et al. reported that 206 of 2703 sera (7.6%) from Japan were positive for PRCV antibodies. Antibodies against PRCV were detected in pigs in all six regions studied [41]. A recent study performed by Kim et al. [44] in South Korea showed that the seroprevalence of PRCV was 41.1% (144/350). High seroprevalence rates for PRCV, 53.1% and 63.7%, were also previously reported in Korea when sera collected in 1999 from 24–26-week-old breeding pigs [9] and sera collected in 2009 were tested [8]. In 2024, PRCV RNA was detected in 28.3% (17/60) of oral fluid samples in the country [44]. A fairly high seroprevalence of 8.7% was also detected in wild boars tested in this country in 2011 [8]. These results indicate that PRCV has been circulating among Korean pigs at a high prevalence since the 1990s.

Jabrane et al. revealed that 74.8% of serum samples from five- to six-month-old pigs in Quebec were seropositive for PRCV [33]. The current study yielded the opposite results. Among the 1245 lung samples collected in 2022 in the U.S., only 0.4% (5/1245) were positive for PRCV [35]. No antibodies to PRCV were detected in the West Indies when 309 pig samples were randomly selected for serological testing [43]. Antibodies against PRCV were also not detected in any of the peccaries or wild boars tested from samples collected between 2014 and 2017 in Argentina [37] or in any of the 93 samples from wild boars from northern Turkey collected during the 2012 hunting season [42].

Main gaps and challenges:

1. Underreporting of PRCV: Prevalence studies are outdated or limited to specific regions, making it difficult to assess the current global incidence of PRCV. Many PRCV infections are mild or subclinical, leading to underreporting of PRCV cases.
2. Regional Variability: Some countries report high seroprevalence of PRCV, but exact infection rates and transmission dynamics remain unclear.
3. Misdiagnosis: Serological tests do not always differentiate PRCV from TGEV, leading to potential misinterpretation of results.
4. Wildlife Reservoirs: The role of wild boars as potential reservoirs for PRCV is not well examined.
5. Coinfections with Other Respiratory Pathogens: PRCV often occurs in combination with other respiratory pathogens, such as PRRSV, *Actinobacillus pleuropneumonia* and *Mycoplasma hyopneumonia*. The role of co-infection in disease progression and severity is an area that requires more in-depth research.
6. Cross-protection against TGEV: Exposure to PRCV may induce partial immunity to TGEV, but the extent and duration of this protection requires further study.
7. Seasonal occurrence: In some herds, PRCV can be isolated from pigs year-round, while in others PRCV temporarily disappears during the summer months, therefore the survival of PRCV in the environment affecting its spread is unclear.

## 3. Molecular Biology of TGEV/PRCV

**Figure 1:**
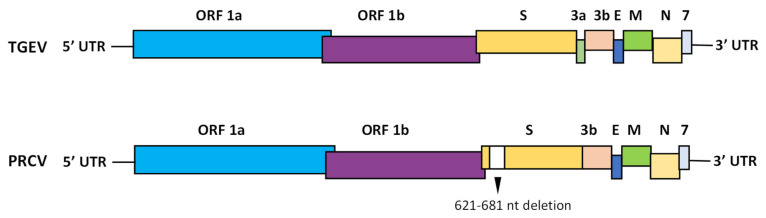
Schematic organization of the TGEV and PRCV genomes. Nonstructural proteins including open reading frame 1a (ORF1a), open reading frame 1b (ORF1b), open reading frame 3a (3a), open reading frame 3b (3b) and open reading frame 7 (7). Structural proteins including spike (S), envelope (E), membrane (M) and nucleocapsid (N). The genome is flanked by 5′ and 3′ UTRs (untranslated regions). The most obvious difference between PRCV and TGEV is a deletion of variable size (621–681 nt) within the amino-terminal segment of the S gene.

TGEV and PRCV are enveloped viruses with a single-stranded positive-sense RNA genome with a 5′ cap structure and a 3′-polyadenylated tail. The TGEV genome is approximately 28.5 kb long, whereas the PRCV genome is 28 kb in size. The PRCV genome has a large deletion in the spike gene and minor deletions in the ORF3a gene (Figure 1). The genome includes nine open reading frames (ORFs) that encode four structural proteins (ORF2, ORF4, ORF5 and ORF6) and five nonstructural proteins (ORF1a, ORF1b, ORF3a, ORF3b, and ORF7) and is organized as 5′ UTR-ORF1a/b-S-ORF3a-ORF3b-E-M-N-ORF7-3′ UTR. A transcriptional regulatory sequence (TRS) is found upstream of each gene. The TRS contains a core sequence, 5′CUAAAC3′, that is highly conserved among coronaviruses [61].

Two-thirds of the virus genome contains ORF1a, and ORF1b encodes polyprotein 1a (PP1a) and polyprotein 1b (pp1b). ORF1a/b are divided into 16 ORFs, which encode the nonstructural proteins Nsp1-10 and Nsp12-16, which are involved in genome replication/transcription [61,62]. Nsp1 is a protein that is expressed early by the virus in the host and interferes with the host’s innate immune response (type I IFN system); thus, it may play a role in the pathogenicity of coronaviruses [63,64]. Nsp2 is a major protein involved in the activation of the NF-KB pathway, which induces inflammation during TGEV infection [65]. Nsp3 is associated with protease and ADP-ribose 1′′-monophosphatetase activities [66,67]. Nsp4 participates with Nsp3 in the generation of double-membrane vesicles (DMVs) during infection [68]. Nsp5 exhibits protease activity and may be involved in interferon antagonism [69]. Nsp6 may be engaged in the formation of double-membrane vesicles [70], whereas Nsp7-16 interact with each other and probably form protein complexes involved in viral replication and transcription [71]. Nsp2, 3, 8 and 12 of TGEV can be incorporated into viral particles [72].

**Figure 2:**
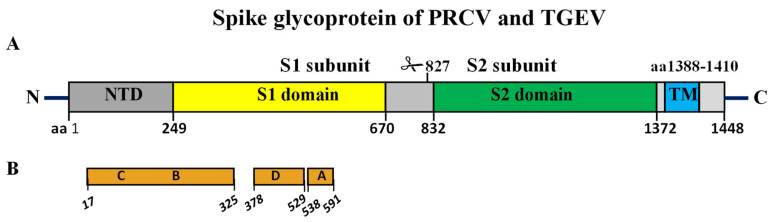
Schematic organization of the amino acids of the TGEV and PRCV spike glycoproteins. (**A**) Domain structure of the TGEV S glycoprotein, NTD (aa 1–248), S1 domain (aa 249–670), S2 domain (aa 832–1372), TM (aa 1388–1410) and C-terminal cytoplasmic tail (aa 1411–1448). (**B**) The relative positions of known antigenic sites (A–D) containing neutralizing epitopes in the spike glycoprotein of TGEV [79].

One-third of the virus genome encodes structural proteins, including S, E, M and N and three accessory proteins, NS3a, NS3b and NS7, encoded by ORF3a, ORF3b and ORF7, respectively. It was suggested that deletion in the ORF3a gene may lead to attenuation and reduced pathogenesis in TGEV [73]. ORF7 encodes a protein located intracellularly, which may play a role in membrane integrity during viral replication and/or viral assembly [74]. The M protein is the main viral membrane protein located in the lipid envelope and is involved in virion assembly in the Golgi via virus nucleoprotein and genomic RNA. The E protein is also a transmembrane protein and is involved in coronavirus maturation. This protein is highly expressed inside infected cells, but only a small fraction of this protein is incorporated into the viral envelope. Therefore, the E protein may have additional functions in the Golgi region and the host cell’s endoplasmic reticulum [75]. The N protein is a structurally phosphorylated protein that interacts with viral RNA, forming a ribonucleoprotein to protect the genomic RNA. Furthermore, it interferes with interferon signaling through different mechanisms [76,77,78]. The spike gene of TGEV is approximately 4.3 kb in length. The S protein is the main protein of the viral envelope that binds to the host cellular receptor aminopeptidase N (APN) or sialic acid (SA). The S protein contains five domains: the NTD at the N-terminal site, the S1 and S2 domains, the transmembrane domain TM and the C-terminal cytoplasmic tail (Figure 2) [79].

The S1 subunit specifically recognizes cellular receptors, whereas the S2 subunit is involved in membrane fusion. PRCV utilizes APN as a receptor for cell entry, while TGEV can additionally bind to SA. TGEV can bind two sialic acids, N-acetylneuraminic acid and N-glycolylneuraminic acid, and bind to these sialic acids through the NTD domain in the S protein [80]. The region of the TGEV S protein that is engaged in binding to SA is located in coding sequences that are absent in the PRCV gene [81]. Therefore, the PRCV does not show intestinal tropism. Furthermore, the S protein is involved in cellular fusion, stimulates neutralizing antibodies that block virus attachment to cells and has hemagglutination activity [34]. The palmitoylated cysteine-rich motif (CRM) situated at the carboxyl end of the TGEV S glycoprotein facilitates the assembly of the S protein during the maturation of virus particles [82].

Four major antigenic sites, A–D, have been characterized at the N-terminal end of the TGEV S protein. The pAPN-binding domain of the S protein has two major antigenic sites (A and B) that have been mapped. Sites A and B contain epitopes that depend on glycosylation. Sites C and D contain glycosylation-independent linear epitopes [83]. There is a loss of one or two antigenic sites in PRCV strains because of deletions in the 5′ region of the S gene.

Main gaps and challenges:

1. Since PRCV is a mutant of TGEV, the functions of PRCV encoded proteins are primarily inferred from TGEV studies rather than studied directly.
2. PRCV likely uses APN as a receptor, similar to TGEV, but how the truncated S protein affects binding affinity and viral entry is not fully understood.
3. The role of the nucleocapsid (N) protein in RNA genome packaging and its interactions with membrane (M) and envelope (E) proteins needs further elucidation.
4. While some nsps have been studied in other coronaviruses, the function of TGEV/PRCV nsps is not well studied.
5. The exact role of accessory genes (ORF3, ORF4, and ORF7) needs further investigation.
6. The molecular details of interactions between the structural and nonstructural proteins of PRCV/TGEV with host immune factors, such as interferon responses and neutralizing antibodies, remain poorly understood.

## 4. Genetic and Phylogenetic Diversity of TGEV and PRCV

### 4.1. TGEV

On the basis of molecular and phylogenetic analysis, TGEV can be divided into two distinct genotypes, traditional (I) and variant (II), with genotype I further divided into the Purdue (Ib) and Miller (Ia) subtypes [14,34,43]. The Purdue subgroup includes the Purdue 115, WH-1, AYU, SC-Y, SHXB and AHHF strains, whereas the Miller subgroups include JS2012, H16, TS, Attenuated H, CN12, Miller 6 and Miller 60. A sequence analysis used to analyze the global evolutionary relationship revealed that the traditional (I) TGEV group consisted of TGEV strains isolated from the U.S., China, South Korea, England, Mexico, Vietnam and Japan, whereas the variant (II) TGEV group consisted of TGEVs from the U.S. collected between 2006 and 2014 [34,84,85,86]. However, complete sequences of TGEV in public databases are limited. The variant (II) TGEV is the dominant genotype circulating in the U.S. Rawal et al. [35] revealed that U.S.-variant TGEV strains share similar changes (nt mutation and aa deletion) with recently isolated PRCV strains, suggesting the occurrence of recombination between variant TGEV and PRCV. Chen et al. [34] revealed eight unique deletions that are present in U.S. variant strains and missing in traditional TGEV strains (three deletions in ORF1a, two deletions between ORF3a and S genes, one deletion in ORF3a, one deletion between ORF3a and ORF3b genes and one deletion in the M gene) [33].

As demonstrated by Cheng et al. [87], genotype-specific codon usage was observed in TGEV strains. In addition, the authors suggested a greater efficiency of protein expression in the hosts infected with the traditional TGEV strains (Ia and Ib). Thus, these strains may be more adapted to the host pig than the variant (II) TGEV strains.

### 4.2. PRCV

Through phylogenetic analysis, the PRCV strains were divided into two groups, the European and American groups, on the basis of geographic distribution, and it was suggested that each PRCV group originated from a different ancestry. This division of PRCVs into American and European strains was evident in trees developed on the basis of whole-genome sequences and for each of the genes encoding structural proteins, with the exception of a tree based on the E gene [26]. Interestingly, Korean PRCV strains have been grouped into a European clade [14,44]. It has been suggested that the European and Korean PRCV strains are closely related to traditional TGEV strains (Miller subgroup) and thus may share a common ancestor. However, these strains clustered separately from the TGEV strains on the phylogenetic tree [4,14]. American PRCV strains are more similar to variant TGEV strains than to European PRCV strains, and it is likely that they evolved from different ancestors [26]. On the other hand, the 5′S gene sequences of the Canadian PRCV strains were most similar to traditional TGEV strains belonging to the Miller subgroup (Ib) [14].

The size of the deletion in PRCV varies from 621 to 681 nucleotides (nt), depending on the origin of the strains. It is widely believed that a deletion in the S gene plays a major role in the altered tissue tropism of PRCV. Most PRCV strains from Europe and Korea have an identical 672 nt deletion at the same position at the 5′ end of the S protein, indicating that they are derived from the same precursor. PRCV strains from the U.S. have deletions of different sizes located at different positions. Some sequences that had a 648 nt deletion in the S gene starting from nt 106 to nt 753 were detected, as were sequences that had a 681 nt deletion starting from nt 64 to nt 744 and sequences that had a 675 nt deletion starting from nt 58 to nt 732 [15]. In addition to PRCV strains with a 672 nt deletion, PRCV strains with a 690 nt deletion which differ in size and location from the European, Korean and American strains have been detected in Poland. This may indicate that different PRCV strains coexist in Poland and possibly originated from different ancestors [14]. Furthermore, Lőrincz et al. [19] detected PRCV strains in Hungary which had a 672 nt deletion in the S gene typical of European strains, but in addition, they detected PRCV strains with a 975 nt deletion. Initially, nine amino acid residues were identified as unique to European PRCV sequences (residues S^307^, L^319^, V^362^, A^435^, S^457^, I^538^, K^578^, T^579^, and P^583^), but when more sequences were included in the analysis, some of them appeared to be not conserved [4]. The isoleucine at position 538 in the S protein was initially found only in European PRCV strains, but newer sequences of European PRCV strains had valine at this position, as did the U.S. PRCV [4]. Moreover, most European PRCV strains had a threonine at position 579, but newer PRCV sequences obtained from Denmark had an isoleucine at this position. Both threonine and isoleucine were found at position 579 in American PRCV strains [4].

Main gaps and challenges:

1. Most phylogenetic studies focus on the spike (S) gene or other viral genes rather than full-genome sequences.
2. The availability of full-genome sequences from different geographical regions and time points is scarce, limiting accurate phylogenetic studies and understanding of the long-term evolution of TGEV and PRCV. While the majority of the TGEV/PRCV sequences are from Europe, North America and China, sequences of these strains from, e.g., Africa and South America are missing or very limited.
3. PRCV is believed to have evolved from TGEV via a large deletion in the S gene, but the exact timeline and geographic origin of this event remain uncertain.
4. Specific mutations defining TGEV variants (traditional versus variant; Purdue versus Miller) are not well documented.
5. The genetic differences between U.S. and European PRCV strains have been observed, but the specific mutations that define these regional variants are not well characterized. More phylogenetic and molecular analyses are needed to confirm these regional differences.

## 5. Molecular Features Related to Virulence and Tissue Tropism

Deletions in the S and ORF3 genes are generally thought to be associated with changes in tissue tropism and virulence. The S genes of the traditional TGEV strains were 4344, 4347 or 4350 nt long, whereas the S genes of the variant TGEV strains were 4350 nt long. Compared with the variant TGEV strains, traditional strains with 4344 nt in the S gene had a 6 nt deletion (TATGAT), whereas traditional strains with 4347 nt had a 3 nt deletion (GTT) [35]. The 6-nucleotide deletion at positions 1123–1128 corresponds to the loss of two amino acids, tyrosine (Y) at position 375 and aspartic acid (D) at position 376, in the spike protein. The presence of a 6 nt deletion in the S gene is considered a characteristic feature of TGEV strains belonging to the Purdue subgroup (Ib), represented by the attenuated Purdue strain P115. This strain was obtained by serial passaging (115 passages) on the ST cells of the original TGEV strain, resulting in the deletion of the viral genome [88]. This 6 nt deletion was not detected in highly pathogenic AHHF and JS2012 strains [45], which may suggest that this deletion may play a role in TGEV attenuation. However, not all strains carrying the 6 nt deletion are attenuated [35]. The exceptions are the SHXB and HQ2016 strains isolated from China, which are considered lethally virulent, and the Chinese HX strain, which causes diarrhea [43,52,89].

A 3 nt deletion (nt 2386–2388) in the S gene resulted in aa 796 V deletion in attenuated TGEV strains, but not in virulent TGEV strains. However, this deletion was detected in the pathogenic AHHF strain but was not detected in the pathogenic SHXB and HQ2016 strains. Therefore, this deletion is unlikely to affect the virulence of TGEV, as this deletion has been found in both virulent and attenuated strains [35]. Furthermore, a 2 aa deletion in the S gene (N375_D376) was detected in a VET-16 strain isolated from Vietnam that caused only mild diarrhea in piglets, attenuated Purdue P115 and NEB72-RT strains and recombinant TGEV strains with reduced replication rates in the gut. However, the role of this deletion in virulence has not been confirmed [90].

A 224 aa deletion (positions 1164–1388 depending on the strain) in PRCV corresponding to the sialic acid-binding domain at the N-terminus of the S protein of TGEV was suspected to be responsible for the loss of intestinal tropism of PRCV, since the loss of this domain results in the inability to bind to sialic acid and effectively infect the gastrointestinal tract [80,91]. However, a recent study performed by Wang et al. [85] has not confirmed this. The authors confirmed that the N-terminal domain in the S protein of TGEV is important for virus replication and infection and affects virus virulence but not the tropism of TGEV. The TGEV clone with the 224 aa deletion presented obvious intestinal clinical manifestations but lower mortality in experimentally infected piglets than TGEV without the deletion, indicating that the N-terminal domain of the S protein is not an exclusive determinant of intestinal tropism. Therefore, it has been speculated that other genes, in addition to the 224 aa N-terminal S protein of TGEV, may regulate TGEV tissue tropism [85].

Some studies have suggested the potential involvement of ORF3 genes in virus virulence [92,93]. A previous study revealed that compared with the TGEV strains of Purdue, the Miller subgroup and variant TGEV strains had a deletion of 29 nucleotides (nt) before the TAA stop codon in the ORF3a gene and a 16 nt deletion before the ATG initiation codon in the ORF3a gene, which may be helpful in distinguishing between the Miller and Purdue subgroups [45,52,94]. These deletions were not found in pathogenic TGEV AHHF, SHXB or HQ2016 strains [35,52,95] but were observed in the Chinese recombinant strain JS2012, which caused significant mortality in piglets, indicating that these deletions are not necessary for viral virulence [45]. Furthermore, a 29 nt deletion in the ORF3 gene has been detected in the PRCV-ISU-1 strain. However, the start and stop codons of ORF3a vary in different PRCV strains, and some strains do not have this gene [35]. The TGEV strain VET-16 from Vietnam, which causes mild diarrhea in piglets, has a large deletion (725 nt) in the ORF3 gene, resulting in the truncation of the ORF3a and ORF3b proteins. Different deletions within the ORF3 genes were observed in TGEV strains, which may suggest that some of them are associated with PRCV [49]. Therefore, it has been suggested that deletions in the ORF3 gene are associated with TGEV attenuation. However, Sola and colleagues demonstrated that deletion of the ORF3 gene in TGEV has very little effect on its infectivity, replication and virulence. The TGEV infectious cDNA clone without the ORF3 gene showed only slightly reduced pathogenicity in vivo but normal replication in cell culture [90]. Therefore, the role of ORF3a/b in viral replication and pathogenesis is still unclear.

The region between the S and ORF3a genes of traditional TGEV strains was 118 or 102 nt in length, whereas this region in variant TGEV strains was 99 nt in length. The ORF3a of the traditional TGEV strains was 216 or 219 nt in length and 219 nt in length in the variant TGEV strains, with the exception of the IL139/2006 strain. The stop codons of ORF3a are not in the same position for all TGEV strains [35].

Not only deletions but also point mutations affect virus virulence and tropism. In attenuated TGEV viruses, but not in virulent strains, a specific mutation (T to G) was found at 1753 nt at the major A/B antigenic sites in the S gene, resulting in a substitution of serine for alanine at position aa 585. This mutation may significantly affect receptor binding or virus interaction with neutralizing antibodies, weakening the virus’s virulence [45]. Alanine, rather than serine, was observed in the PRCV strains, suggesting that it may be an attenuation marker for the TGEV and PRCV strains [96]. This mutation may be involved in reducing the virulence of TGEV strains, but some strains carrying this mutation, such as strains AHHF, SHXB and HQ2016, have been shown to be virulent [52]. Therefore, the importance of S585A loci in attenuation should to be proved by reverse genetics followed by testing in vivo. The presence of Chinese TGEV strains with and without this mutation suggests that the antigenicity of protein S of Chinese TGEV strains may be altered [47]. In addition to the mutations at position 585, mutations at positions 72 and 219 in the S protein of TGEV are determinants of intestinal tropism [97,98]. It has been suggested that the substitution of aspartic acid (D) with asparagine (N) at position 72 and alanine (A) with serine (S) at position 219 are associated with attenuation and loss of intestinal tropism [86,97]. These mutations are located in the fragment of S gene which is deleted in PRCV strains, suggesting that are essential for maintaining intestinal tropism [97]. Using infectious TGEV cDNA and enteric (TGEV-SC11) or respiratory (TGEV-SPTV) isolates, Sanchez et al. [99] confirmed that an S219A change in the S protein resulting in the elimination of a predicted glycosaminoglycan (GAG) attachment site was required to confer intestinal tropism to respiratory TGEV. The Vietnam VET-16 strain which causes mild diarrhea in piglets had asparagine at position 72, serine at position 219 and alanine at position 585, 3 aa changes (V378L, S379T, D380N) and a 9 nt (3 aa) insertion in antigenic site D (aa 378–392). These mutations may change the epitope structure of antigenic site D, which can lead to changes in antigenicity and virulence. Additionally, this strain had a large deletion in the ORF3 gene [86]. Galan et al. reported that a G-to-A mutation at position 637 resulting in a substitution of glicyne (G) for valine at position aa 108 in the ORF1 of TGEV causes cleavage via a papain-like protease, which results in virus attenuation. The effect of this point mutations was analyzed by reverse genetics using a TGEV full-length cDNA clone and cDNAs from TGEV-derived minigenomes [100]. Using a CRISPR/Cas9-based reverse genetics system, Shen et al. [101] showed that recombinant TGEV, in which amino acids 91–95 in nsp1 were altered, did not affect virus replication but significantly reduced TGEV pathogenicity in piglets, suggesting that nsp1 is essential for virulence. Furthermore, Cruz et al. [102] showed that the lack of the gene encoding protein 7 led to an increased pro-inflammatory response and acute tissue damage after infection.

The effects of main deletions and point mutations on virulence and tissue tropism are shown in Table 1.

The different pathogenicities of the PRCV strains are related to changes in the S and ORF3a genes. Keep et al. [103] reported that European strains 135 and 137 were more virulent than the US 310 and ISU-1 strains, but Halbur et al. [104] reported that the US 310 and LEPP strains were more virulent than strains from Europe. A recent study by Rawal et al. [84] revealed that nasal RNA excretion in pigs experimentally infected with the U.S. PRCV variant strain (AR310 from 1989) was significantly greater than that in pigs infected with a traditional U.S. PRCV strain isolated in 2020 (ISU20). This may suggest that the virulence or transmissibility of variant PRCV strains circulating today may be greater than that of traditional PRCV strains [84]. In addition, significantly higher mean PRCV antibody titers were observed in pigs infected with the variant PRCV strain than in pigs infected with the traditional PRCV strain, but no significant differences in microscopic or macroscopic lung lesions were observed between these two groups of pigs [84].

A comparison of PRCV sequences obtained from feces and nasal swabs revealed a difference of 4 nt, but the possibility that the change in tropism was a consequence of these mutations was unlikely [15]. The authors revealed that the fecal-derived PRCV strain 12–19 F lost respiratory tropism when pigs were inoculated intranasally. Furthermore, it was suggested that the loss of respiratory tropism could be caused by two mutations at positions 790 and 791 causing a change from threonine to valine in the aa sequences [15]. This assumption is based on the fact that threonine at the same position was found in the TGEV strain PUR46MAD, which has intestinal and respiratory tropism, and in the PRCV strain ISU-1, which has only respiratory tropism [15].

In addition to randomly generated mutations and deletions, RNA recombination of coronaviruses simultaneously infecting a given host may occur. This can lead to the emergence of new coronaviruses with new biological properties, such as altered pathogenicity, tissue tropism and host specificity. The highly pathogenic TGEV strain JS2012 is a natural recombinant of Miller M6 and Purdue P115, i.e., TGEVs belonging to the same group, whereas the TGEV strain AHHF is a recombinant of TGEV strains belonging to genotypes I (traditional) and II (variant) [45,46,79]. The AHHF strain was highly virulent, although it had mutations previously thought to cause attenuation [35]. The presence of strains that have attenuation-related mutations but strong virulence is surprising. Not only intraspecies recombination occurs. Co-infection with various porcine enteric coronaviruses is also common. Some of these viruses can infect the same cells, providing beneficial conditions for recombination. Recently, a new chimeric virus resulting from the recombination of PRCV/TGEV with PEDV, called porcine enteric coronavirus (SeCoV), has been reported in several European countries, including Spain, Germany, Italy and Slovakia [105,106,107]. Most of the genome of this new virus is derived from TGEV/PRCV, whereas the S gene is derived from PEDV. Recombination was detected at positions 20,636 and 24,867 of PEDV and at positions 20,366 and 24,996 of TGEV [107]. This virus causes diarrhea, vomiting and severe dehydration in young piglets, resulting in high mortality rates. The detection of SeCoV in samples from diarrhea outbreaks in Spain in 1993 suggests that the recombination between the PEDV and TGEV occurred a long time ago [108]. Recombination was also observed between swine acute diarrhea syndrome coronavirus (SADS-CoV) and TGEV. This TGEV-like strain has integrated S gene sequences derived from SADS-CoV [109]. Thus, in both new viruses, recombination breakpoints were located within the S gene. However, Guo et al. observed that the recombination frequency of TGEV was lower than that of other swine enteric coronaviruses, such as porcine deltacoronavirus (PDCoV) and swine acute diarrhea syndrome coronavirus (SADS-CoV). It was also indicated that interspecies recombination is most likely between coronaviruses belonging to the same genus than between viruses from different genera [109].

TGEV mainly infects pigs, but cats, dogs and foxes can be carriers of TGEV [110]. TGEV has been isolated from dogs showing severe diarrhea in China [111]. Canine coronaviruses containing the N-terminal domain of the S gene of TGEV (TGEV-like CCoV) were detected in the feces and intestinal organs of naturally infected dogs that died of acute gastroenteritis [112]. TGEV excreted by infected dogs has been confirmed to be infectious to pigs. It has been suggested that TGEV, PRCV and canine and feline coronaviruses are derived from the same coronavirus ancestor [113]. All of these coronaviruses are genetically, biologically and antigenically closely related. They cross-react with monoclonal antibodies directed against proteins N, S and M in a virus neutralization assay, and all share antigenic site A on protein S. Furthermore, TGEV, PRCV and FCoV use the same receptor, aminopeptidase N [113]. Recently, Vlasova et al. [114] characterized the complete genome of the canine-feline recombinant alphacoronavirus (CCoV-HuPn-2018, GenBank accession no. MW591993.2), which was isolated from a child with pneumonia in Malaysia. Interestingly, the sequence of this strain was 90% similar to that of the TGEV genome of Purdue. In addition, Lednicky et al. [115] isolated a highly similar recombinant coronavirus from a member of the medical team who had fever and malaise after a trip to Haiti, and this strain in a phylogenetic tree on the basis of the N gene cluster with TGEV. These findings only highlight the possible zoonotic risk of TGEV. Unquestionably, further studies are needed to investigate the pathogenic potential of TGEV in humans.

Main gaps and challenges:

1. Specific deletions or mutations in TGEV/PRCV have mostly been identified in comparative studies, and their actual functional impact is often hypothetical but not experimentally confirmed using reverse genetics systems. This is a major knowledge gap that limits our understanding of how specific genetic changes affect virulence and tissue tropism.
2. ORF3 has been suggested to play a role in viral replication and virulence, but its exact function remains poorly defined. The exact contribution of other structural and nonstructural proteins to TGEV/PRCV virulence remains poorly characterized and requires further research.
3. Specific mutations defining TGEV variants (traditional versus variant; Purdue versus Miller) are not well documented.
4. The exact genetic or molecular differences that make some PRCV strains more virulent than others are not well understood. European and U.S. PRCV strains may differ in genetic makeup and virulence, but detailed comparative studies are lacking.
5. Comparing PRCV sequences from feces and nasal swabs could provide insights into viral replication sites, transmission dynamics and potential dual tropism. However, significant knowledge gaps exist in this area, limiting our understanding of PRCV pathogenesis and evolution.
6. Intraspecies recombination between different TGEVs and recombination between TGEV and other enteric viruses raises concerns about the emergence of new, highly pathogenic strains. However, there are significant gaps in knowledge about the frequency, mechanisms and consequences of these recombination events. More whole-genome sequencing of field strains is needed to identify recombination events in pig populations.
7. The relationship between TGEV/PRCV and other alphacoronaviruses (e.g., feline coronavirus, canine coronavirus) needs deeper analysis.
8. Limited information exists on the potential impact of TGEV on human health, either directly or indirectly through zoonotic transmission or as a source of genetic material for coronaviruses infecting humans. Understanding any potential human health risks associated with TGEV is a key area for further research.
9. Although PRCV is closely related to TGEV, its potential to infect other species, including humans, has not been studied.

## 6. Conclusions

The occurrence of TGEV has been very low in recent years, but there are still countries where TGEV is more prevalent. The low incidence of TGEV is believed to be related to the spread of PRCV. However, recent studies have reported different detection rates of PRCV, suggesting that other factors may contribute to the decline in TGEV. However, it is worth noting that comparing prevalence as a raw number is difficult because it does not consider factors such as population size, the sensitivity of the test used, the age of the animals tested and the presence of other pathogens. Therefore, the real prevalence of TGEV and PRCV is underestimated. In addition, recent epidemiological studies of TGEV and PRCV in many countries are lacking. The low prevalence of TGEV indicates that PRCV does not completely protect against TGEV infection. Positive TGEV samples were found on several PRCV-positive farms. In addition, TGEV outbreaks have been observed in Japan, England and Hungary on farms with simultaneous TGEV and PRCV infection [19,41]. An explanation for this may be that PRCV-induced antibodies are detectable for approximately one year after infection [116]. Thus, after this time, the animals can be reinfected with TGEV. PRCV infection induces antibodies that cross-react with TGEV. However, PRCV infection leads to the production of neutralizing antibodies to antigenic site A within the S protein but not the D site. Therefore, it is possible that a lack of site D reactivity is a reason why an earlier PRCV infection does not provide full cross-protection against TGEV. It was also shown that pigs induced lactogenic antibodies after PRCV infection, which protected against TGEV infection, but to a lesser degree than TGEV-induced antibodies [117]. The presence of other pathogens that can enhance TGEV pathogenicity during a TGEV outbreak (*E.coli*, rotavirus) cannot be excluded. In addition, the genomes of PRCV and TGEV are constantly evolving. Changes in proteins related to immunogenicity, neutralization, receptor binding capacity, virulence activity and tropism can alter the host immune system’s ability to recognize the virus and the virus’s efficacy at inducing clinical disease. Mutations in the receptor binding domain (RBD) of the S protein may reduce the ability of antibodies to bind effectively. This could result in partial immunity to certain strains, meaning that pigs may be susceptible to reinfection or infection with variants of TGEV. Mutations in other structural and nonstructural proteins also play roles in the modulation of the host’s immune response. For example, it was indicated that substitution of threonine to isoleucine at position 17 in the M protein resulted in lower IFN-α induction as compared to the wild-type virus, which may lead to a lack of induction of the antiviral state in host cells, leading to more efficient replication and immune evasion [118]. Moreover, amino acid residues at positions 590–1215 in Nsp3 of TGEV form a critical domain that mediates nuclear factor-kappa B’s (NF-κB’s) inhibition of the host immune system [119]. It has also been shown that the first 120 amino acid residues of the Nsp2 TGEV protein play a key role in activating NF-κB and increasing the expression of pro-inflammatory cytokines, so mutations at this site may alter the virus’s ability to modulate the immune response [65]. To conclude, the degree of cross-protection can vary depending on factors such as the specific viral strains involved, the immune status of the pig, and the presence of other pathogens. In addition, recombination is responsible for the recent emergence of novel genetic strains of TGEV, some of which have more pronounced pathogenic potential. Recombination between TGEV and other coronaviruses has also led to the emergence of new chimeric viruses, such as SeCoV or TGEV-like CCoV, which were recently identified in pigs and dogs, respectively, in Europe. Therefore, continuous monitoring, genome sequence analysis and studies of interspecies transmission and host adaptation are critical for understanding genetic evolution of TGEV and PRCV and preventing and controlling TGEV/PRCV-induced diseases.

## Figures and Tables

**Table 1:** The effects of main deletions and point mutations on virulence and tissue tropism.

Strain	Isolate	Variation	Site	Effect	References
Purdue	Purdue P115, WH-1, HX, SC-Y, PUR46-MAD, SHXB, HQ2016	6 nt deletion (TATGAT) at position 1123–1128 (aa deletion of Y at position 375 and D at position 376)	spike	Reduced virulence/viral attenuation (Exception: SHXB, HQ2016, HX)	[45,46]
Miller	Attenuated H, H16, AHHF, Miller M60	3 nt deletion (TTG) at 2387–2389 (aa 796V)	spike	Unclear effect; found in both virulent and attenuated strains	[46]
-	TGEV mutant	224 aa deletion at positions 17–240 of S protein	spike	* Mildly reduced virulence, no impact on tropism	[85]
Miller	Attenuated H, H16, Miller M60, Miller M6, JS2012, PRCV-ISU-1	16 nt deletion (TCTGCTAGAGAATTTT) and 29 nt deletion (CAATAGTCATATAGTTGTTTAATATCATT)	ORF3a/b	Reduced virulence	[45]
Purdue	PUR46-MAD full-length cDNA clone	872 nt deletion at positions 24,822–25,693	ORF3a/b	* Limited reduction in virulence	[90]
Purdue, Miller	VET-16, H16, Purdue 115, Miller M60, attenuated H, AHHF, SHXB, HQ2016, PUR-46-MAD, WH-1, SC-Y, PRCV-ISU-1	1753 nt T to G (585 aa S to A)	spike	Reduced virulence (Exception: AHHF, SHXB and HQ2016 strains)	[45,73]
Purdue, Miller	VET-16, Miller M6, H16, Miller M60, attenuated H, PRCV	214 nt G-to-A (72 aa D to N)	spike	Reduced virulence, loss of intestinal tropism	[97]
Purdue	VET-16, NEB72-RT, PUR-46-MAD, PRCV	655 nt G to T (219 aa A to S)	spike	Reduced virulence, loss of intestinal tropism	[97,99]
-	TGEV full-length cDNA clone and TGEV-derived minigenomes	637 nt G-to-A (108 aa G to V)	ORF1	* Affected papain-like protease 1-mediated cleavage in vitro	[100]

* The impact of deletions and point mutations on virulence and tissue tropism was based on state-of-the-art reverse genetics, followed by in vivo testing. In other cases, the impact was based on sequence comparison of different viruses (forward genetics).

## Data Availability

Not applicable.
